# Concomitant arthroscopy during valgus‐producing high tibial osteotomy does not demonstrate consistent additional clinical benefit despite improved cartilage findings: A systematic review of comparative studies

**DOI:** 10.1002/jeo2.70903

**Published:** 2026-09-07

**Authors:** Dave Osinachukwu Duru, Ebenezeer Mussie, Taimoor A. Sehgol, Yuta Nakanishi, Ryan M. Degen

**Affiliations:** ^1^ School of Clinical Medicine University of Cambridge Cambridge UK; ^2^ Schulich School of Medicine and Dentistry Western University London Ontario Canada; ^3^ Department of Surgery, Fowler Kennedy Sport Medicine Clinic, Bone and Joint Institute Western University London Ontario Canada

**Keywords:** arthroscopy, high tibial osteotomy, meniscectomy, microdrilling, microfracture

## Abstract

**Purpose:**

To evaluate whether concomitant arthroscopic treatment of intra‐articular chondral or meniscal pathology during valgus‐producing high tibial osteotomy (HTO) improves clinical outcomes compared with HTO alone.

**Methods:**

MEDLINE, Embase and CENTRAL were searched from inception to 17 May 2026. Comparative studies reporting outcomes after HTO plus arthroscopic cartilage (microfracture, microdrilling, abrasion arthroplasty) and/or meniscal (meniscal centralization, meniscectomy, meniscal repair) procedures versus HTO alone were included. Data extraction and quality assessment were performed by two reviewers. Due to heterogeneity, meta‐analysis was not performed, and outcomes were synthesized descriptively, stratified by specific arthroscopic adjunct.

**Results:**

Seventeen studies comprising 1271 knees were included, with 663 treated with HTO plus arthroscopy and 608 treated with HTO alone. The mean age was 57.0 years with HTO plus arthroscopy and 55.1 years with HTO. Mean follow‐up was 32.9 months and 31.0 months, respectively. Concomitant procedures were microfracture in six studies, microdrilling in three, abrasion arthroplasty in two, meniscectomy in two, meniscal centralization in two and mixed arthroscopy in four. Both groups generally improved across patient‐reported outcome measures (PROMs), functional outcomes and radiographic alignment. However, no arthroscopic adjunct demonstrated consistent additive clinical benefit. Microfracture showed inconsistent effects on Lysholm and Western Ontario and McMaster Universities Osteoarthritis Index scores; meniscectomy did not improve Knee Society Score; and meniscal centralization showed inconsistent Knee injury and Osteoarthritis Outcome Score and alignment findings. Mixed arthroscopy showed some favourable PROMs and radiographic outcomes, but did not isolate procedure‐specific effects. Microfracture, microdrilling, abrasion arthroplasty and meniscal centralization improved cartilage findings on second‐look arthroscopy, but these improvements did not consistently correspond to superior PROMs. Complication reporting was limited and heterogeneous.

**Conclusions:**

Current evidence does not demonstrate a consistent clinical benefit of adding arthroscopic chondral or meniscal treatment to HTO, despite some evidence of improved cartilage findings. Future procedure‐specific comparative studies are needed to determine which, if any, arthroscopic adjuncts provide clinically meaningful additive benefit when performed with HTO.

**Level of Evidence:**

Level III.

## Graphical abstract

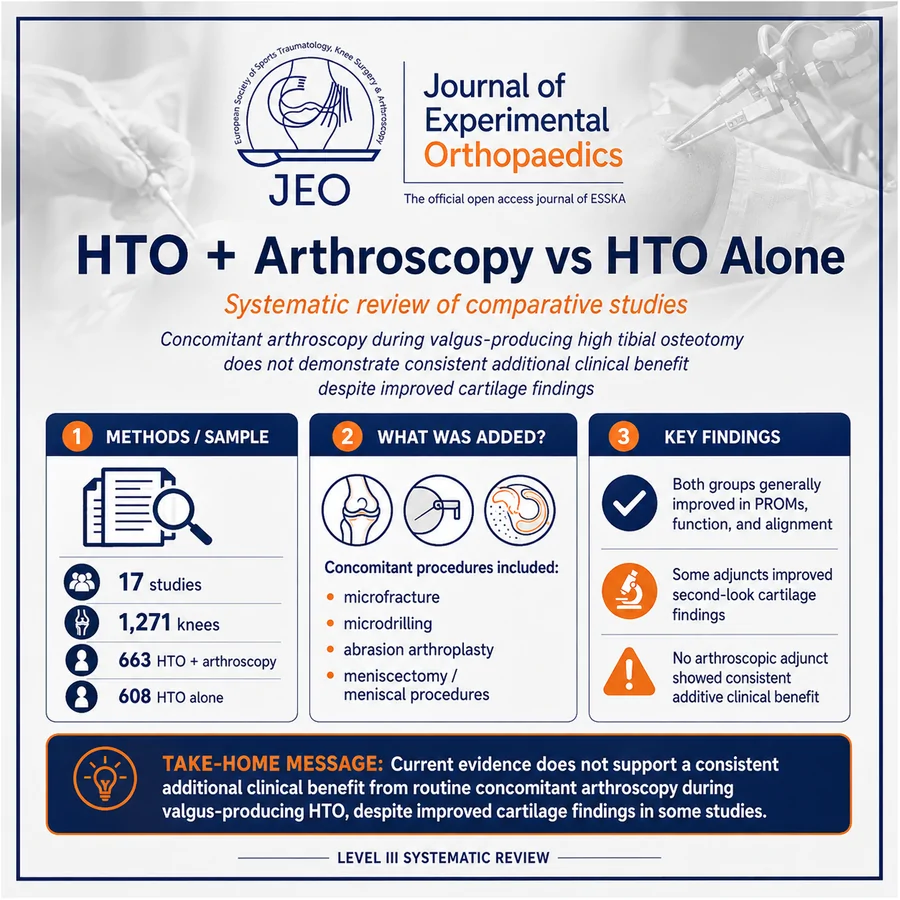

**Abbreviations**
ADL: activities of daily living
BMI: body mass index
CENTRAL: Cochrane Central Register of Controlled Trials
FTA: femorotibial angle
FU: follow‐up
HKA: hip–knee–ankle angle
HSS: Hospital for Special Surgery
HTO: high tibial osteotomy
IKDC: International Knee Documentation Committee
IQR: interquartile range
JLCA: joint line convergence angle
JOA: Japanese Orthopaedic Association
KOOS: Knee injury and Osteoarthritis Outcome Score
KSS: Knee Society Score
LOE: level of evidence
MCID: minimal clinically important difference
MeSH: Medical Subject Headings
mFTA: mechanical femorotibial angle
MINORS: Methodological Index for Non‐Randomized Studies
mLDFA: mechanical lateral distal femoral angle
MOCART: Magnetic Resonance Observation of Cartilage Repair Tissue
MPTA: medial proximal tibial angle
mTFA: mechanical tibiofemoral angle
NR: not reported
PASS: Patient Acceptable Symptom State
PRISMA: Preferred Reporting Items for Systematic Reviews and Meta‐Analyses
PROMs: patient‐reported outcome measures
PROSPERO: International Prospective Register of Systematic Reviews
PTS: posterior tibial slope
QoL: quality of life
RCT: randomized controlled trial
RoB 2: Risk of Bias 2
ROM: range of motion
SD: standard deviation
TFA: tibiofemoral angle
TKA: total knee arthroplasty
VAS: visual analogue scale
WBLR: weight‐bearing line ratio
WOMAC: Western Ontario and McMaster Universities Osteoarthritis Index

## INTRODUCTION

Patients undergoing high tibial osteotomy (HTO) frequently present with intra‐articular meniscal or chondral pathology [22], and arthroscopy may be performed prior to HTO to treat such defects [33]. Common concomitant arthroscopic interventions include meniscectomy, meniscal repair or meniscal centralization to address meniscal lesions and abrasion arthroplasty, microfracture or microdrilling to treat chondral defects [34, 35]. These procedures are distinct from restorative cell‐ or scaffold‐based strategies, such as autologous chondrocyte implantation or osteochondral grafting [18, 31], as they do not biologically augment the interventions and can be performed more routinely at a lower cost. While thought to improve patient symptoms, it remains unclear whether treating meniscal or cartilage pathology during HTO provides clinical benefit beyond the realignment effects of HTO itself. Additionally, concomitant arthroscopy may increase operative time, procedural complexity and resource utilization, while exposing patients to additional procedural complication risks [10, 35]. Therefore, determining whether arthroscopy provides clinically meaningful benefit to HTO is imperative for surgical decision‐making.

Previous reviews have suggested benefits of combining HTO with arthroscopic cartilage procedures [1, 11, 32]. However, existing comparative syntheses have pooled either heterogeneous cartilage procedures [6] or substantially different adjuncts such as meniscal transplantation and injections [20]. Such research designs limit the applicability of the findings to routine arthroscopic procedures performed with HTO and make it difficult to determine which specific arthroscopic procedures may provide additive clinical benefit.

Thus, the purpose of this systematic review was to evaluate whether concomitant arthroscopic treatment of chondral or meniscal pathology during valgus‐producing HTO improves clinical outcomes compared with HTO alone. Outcomes of interest included patient‐reported outcome measures (PROMs), functional outcomes, radiographic alignment, second‐look arthroscopy findings and complications. The hypothesis was that no specific arthroscopic procedure would demonstrate a consistent additive clinical benefit over HTO alone, with respect to PROMs and radiographic alignment.

## METHODS

### Study design

This systematic review was conducted in accordance with the Preferred Reporting Items for Systematic Reviews and Meta‐Analyses (PRISMA) 2020 guidelines [25] to ensure rigour and transparency in reporting. The review protocol was developed a priori and registered with the International Prospective Register of Systematic Reviews (PROSPERO; CRD420251182613).

### Search strategy

A comprehensive literature search was conducted across three databases (Embase, Ovid MEDLINE and Cochrane CENTRAL [Cochrane Central Register of Controlled Trials]) from inception to 17 May 2026. Additionally, the reference lists of the included studies were manually screened to ensure complete article retrieval. The search strategies used a combination of Medical Subject Headings (MeSH) and relevant keywords pertaining to HTO (‘high tibial osteotomy’ or ‘HTO’ or ‘medial opening wedge’ or ‘opening wedge osteotomy’ or ‘closing wedge osteotomy’) and concomitant arthroscopic interventions of the cartilage or meniscus (‘arthroscopy’ or ‘meniscectomy’ or ‘abrasion arthroplasty’ or ‘microdrilling’ or ‘microfracture’ or ‘meniscal centralization’). Full search strategies are detailed in Table S1.

### Study screening

All records were imported into Covidence software (Veritas Health Innovation) for screening and study management. After duplicate removal, two reviewers (D. O. D. and E. M.) independently screened titles and abstracts for relevance. The full‐text articles were retrieved for studies that satisfied the inclusion criteria or in cases of uncertainty. Disagreements regarding study selection were resolved via discussion or consultation with a senior author (T. A. S.). The reasons for exclusion at full‐text screening were documented. The inter‐reviewer agreement was assessed via the kappa (*κ*) statistic, interpreted using standard thresholds: slight (0.00–0.20), fair (0.21–0.40), moderate (0.41–0.60), substantial (0.61–0.80) and almost perfect agreement (0.81–1.00) [19].

### Eligibility criteria

Studies were eligible for inclusion if they were comparative studies evaluating valgus‐producing HTO combined with one or more non‐cell‐based, non‐scaffold‐augmented cartilage or meniscal arthroscopic procedures (e.g., microdrilling, microfracture, abrasion arthroplasty, meniscectomy, non‐root meniscal repair, meniscal centralization or mixture of procedures) compared to HTO alone. Only adult patients (aged ≥18 years) were included. Non‐cell‐based, non‐scaffold‐augmented therapeutic arthroscopy was defined as arthroscopic meniscal or chondral treatment not involving the addition of cells, biologic products or scaffolds to enhance or augment cartilage or meniscal treatment. All HTO surgical techniques were eligible, including both closing wedge HTO and medial opening wedge HTO. Eligible study designs included randomized controlled trials (RCTs) and prospective or retrospective cohort studies. Only studies that reported PROMs, functional outcomes, radiographic outcomes, second‐look arthroscopy findings and/or complications were included. No language restrictions were applied during the search, but studies without an available English full text were excluded.

The exclusion criteria consisted of conference abstracts, technique papers, review articles, textbook chapters, cadaveric studies, case series, case reports and HTO plus arthroscopy cohorts where cell‐ or biologic‐based cartilage repair (e.g., osteochondral autograft/allograft transplantation, meniscal transplantation or scaffold‐based implantation), concomitant ligament reconstruction (e.g., anterior cruciate ligament) or meniscal root repair was performed. Meniscal root repair was excluded because it represents a distinct intervention from conventional repair with a dedicated comparative evidence base [1, 7, 32]. Diagnostic arthroscopy done in isolation was not counted as an arthroscopic intervention, given that no therapeutic procedure was performed on the cartilage or meniscus.

### Data extraction

Data extraction was conducted by two reviewers (D. O. D. and E. M.) using a standardized data collection form in Microsoft Excel (Version 16.100). Extracted variables included study characteristics (author, year of publication, study design, level of evidence, sample size and follow‐up [FU] duration), patient demographics (age, sex), intervention details (HTO technique, concomitant arthroscopic procedure[s]) and clinical outcomes (PROMs, functional outcomes, radiographic alignment outcomes, second‐look arthroscopy findings and complications). Discrepancies were resolved through discussion or consultation with a senior author (T. A. S.).

### Risk of bias and methodological quality assessment

Risk of bias and methodological quality were assessed independently by two reviewers (D. O. D. and E. M.), with disagreements resolved through discussion or consultation with a senior author (T. A. S.). Randomized controlled trials were assessed using the Cochrane Risk of Bias tool for randomized trials (Risk of Bias 2 [RoB 2]), assessing five domains. Overall judgments were classified as low risk, some concerns or high risk of bias. The methodological quality of non‐randomized comparative studies was assessed using the Methodological Index for Non‐Randomized Studies (MINORS) instrument. Each study was scored across 12 domains, with each item scored from 0 to 2 and ascribed an ideal global score of 24 for comparative studies. Studies were classified as poor quality (≤14), moderate quality (15–18) or high quality (19–24).

### Data synthesis and analysis

Given the anticipated clinical and methodological heterogeneity across studies, including differences in study design, patient populations, surgical techniques, concomitant arthroscopic procedures, outcome measures and FU duration, findings were synthesized descriptively, and no meta‐analysis was performed. Descriptive statistics were calculated where appropriate, including weighted mean preoperative and postoperative values for outcomes reported by multiple studies. Where available, ranges of preoperative values, postoperative values and mean changes were reported for each outcome. Given the heterogeneity of reported PROMs and radiographic measures, the narrative synthesis prioritized outcomes reported by at least two studies within the same concomitant arthroscopic intervention subgroup, where possible. Statistical significance was defined as *p* ≤ 0.05.

## RESULTS

### Search results

**Figure 1:**
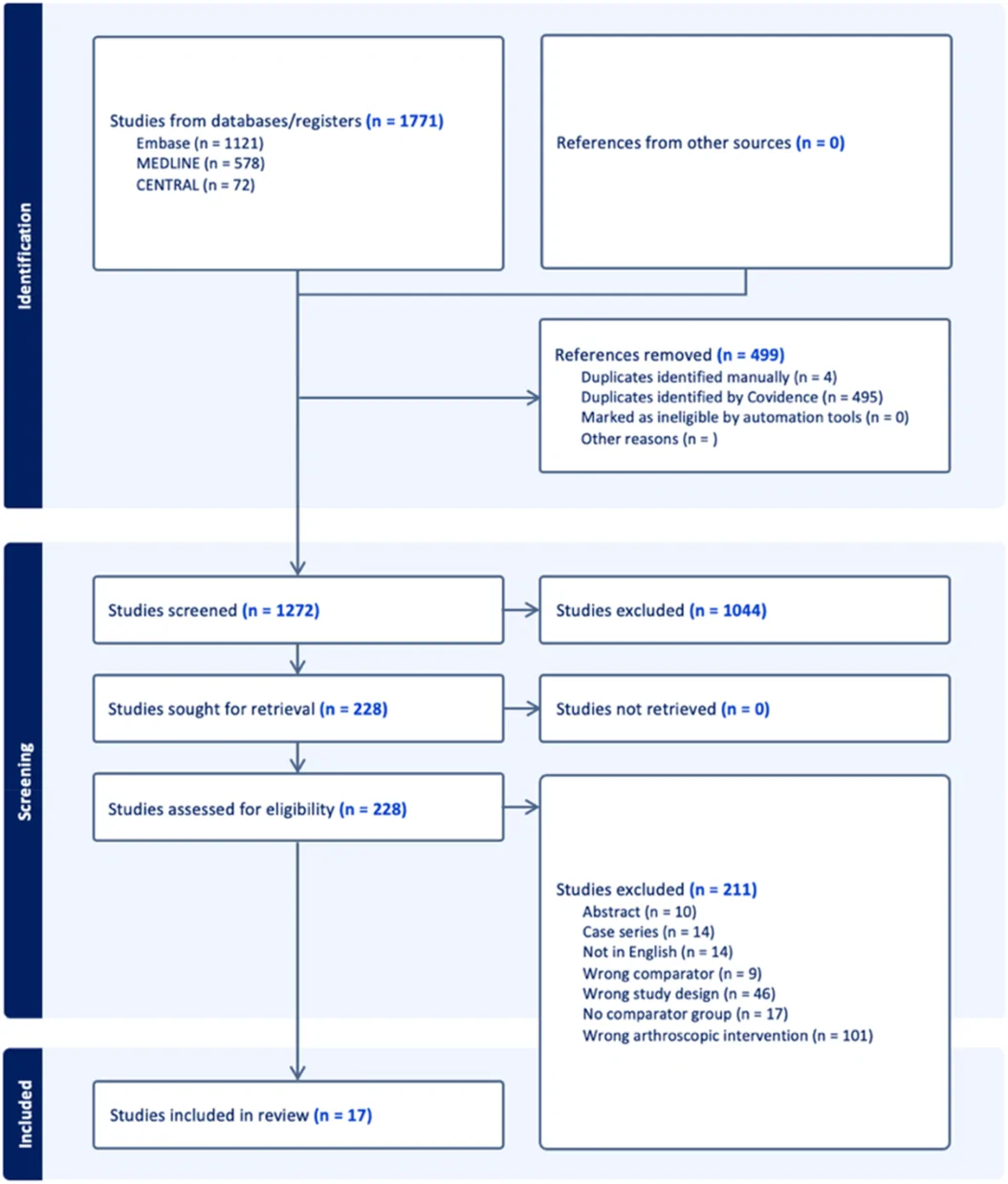
Study selection flowchart based on PRISMA guidelines. PRISMA, Preferred Reporting Items for Systematic Reviews and Meta‐Analyses.

The search identified 1771 potentially relevant studies. After removing 499 duplicates, 1272 records remained for title and abstract screening. The reviewers showed moderate agreement at this stage (*κ* = 0.51). Following exclusion of 1044 articles, 228 full‐text articles were assessed for eligibility, with almost‐perfect inter‐reviewer agreement at this stage (*κ* = 0.85). A total of 211 studies were excluded, leaving 17 studies for the final analysis (Figure 1). Of the included studies, two were RCTs [26, 35], 14 were retrospective cohort studies [2, 4, 5, 9, 12, 13, 14, 15, 16, 21, 23, 28, 30, 34], and one was a prospective cohort study [27] (Table 1).

**Table 1:** Summary of included studies and overall patient demographics.

Author (year)	Study design (LOE)	Intervention	Knees (*n*)	Mean age (SD)	Male (%)	Mean BMI (SD), kg/m^2^	Mean follow‐up (SD), months
[2]	Retrospective Cohort (III)	HTO + microfracture	26	53.2 ± 5.1	5 (19.2%)	26.5 ± 1.5	67.4 ± 5.5
		HTO	24	53.0 ± 4.4	4 (16.7%)	26.5 ± 1.3	67.4 ± 5.5
[9]	Retrospective Cohort (III)	HTO + microfracture	18	Median 53	13 (72.2%)	31 ± 5	Median 92
		HTO	20	Median 54	10 (50%)	26 ± 4	Median 102
[21]	Retrospective Cohort (III)	HTO + microfracture	57	57.0 ± 5.4	37 (64.9%)	26.5 ± 3.6	24.0 ± 2.4
		HTO	30	57.0 ± 6.5	7 (23.3%)	26.4 ± 3.3	22.8 ± 1.2
[23]	Retrospective Cohort (III)	HTO + microfracture	26	65.0 ± 3.8	7 (26.9%)	NR	Minimum 60 months
		HTO + abrasion arthroplasty	51	64.6 ± 5.9	9 (17.6%)	NR	
		HTO	37	64.0 ± 5.9	2 (5.4%)	NR	
[26]	Randomized Controlled Trial (II)	HTO + microfracture	20	50.0 ± 4.6	13 (65%)	26.3 ± 2.9	Minimum 60 months
		HTO	20	49.7 ± 5.8	15 (75%)	28.6 ± 3.7	
[30]	Retrospective Cohort (III)	HTO + microfracture	56	41.4	NR	NR	NR
		HTO	73	39.3	NR	NR	
[14]	Retrospective Cohort (III)	HTO + microdrilling	30	61.5 ± 7.5	3 (10%)	25.8 ± 2.0	25.7 ± 8.3
		HTO	31	58.6 ± 6.9	3 (9.7%)	27.1	24.1 ± 5.7
[13]	Retrospective Cohort (III)	HTO + microdrilling	30	58.7	4 (13.3%)	28.1	32.8
		HTO	30	58	7 (23.3%)	27.1	32
[16]	Retrospective Cohort (III)	HTO + meniscectomy	55	55.6 ± 4.4	13 (23.6%)	25.1 ± 3.1	28.7 ± 9.6
		HTO	24	56.9 ± 4.9	8 (33.3%)	25.5 ± 2.7	33.0 ± 11.1
[5]	Retrospective Cohort (III)	HTO + meniscectomy	Partial: 20 Subtotal: 30	Partial: 56.4 ± 7.1 Subtotal: 58.6 ± 6.1	Partial: 4 (20%) Subtotal: 11 (36.7%)	Partial: 27.0 ± 3.4 Subtotal: 26.9 ± 2.5	Partial: 63.5 ± 14.1 Subtotal: 69.3 ± 15.5
		HTO	9	51.6 ± 9.3	4 (44.4%)	26.3 ± 1.9	64.0 ± 16.2
[12]	Retrospective Cohort (III)	HTO + meniscal centralization	24	63.4 ± 8.1	11 (45.8%)	26.4 ± 3.8	30.0 ± 4.8
		HTO	24	62.0 ± 7.7	10 (41.7%)	26.1 ± 5.2	28.8 ± 6.0
[15]	Retrospective Cohort (III)	HTO + meniscal centralization	21	Median 57	9 (42.9%)	Median 23.5	30.2
		HTO	18	Median 62	7 (38.9%)	Median 24.2	40.7
[27]	Prospective Cohort (II)	HTO + microdrilling	22	64.5 (NR for subgroups)	NR	NR	12
		HTO + abrasion arthroplasty	23		NR	NR	
		HTO	40		NR	NR	
[4]	Retrospective Cohort (III)	HTO + mixed arthroscopy	17	58.8	3 (23.1%)	NR	12.3
		HTO	12	53.2	2 (20%)	NR	24.5
[28]	Retrospective Cohort (III)	HTO + mixed arthroscopy	54	52.5 ± 3.6	NR	NR	19.0 ± 6.2
		HTO	54	53.3 ± 2.2	NR	NR	19.0 ± 6.2
[34]	Retrospective Cohort (III)	HTO + mixed arthroscopy	42	Median 53	21 (60%)	27.1 ± 0.5	Minimum 12
		HTO	121	Median 54	47 (45.6%)	28.0 ± 0.3	
[35]	Randomized Controlled Trial (II)	HTO + mixed arthroscopy	41	58.8 ± 8.2	19 (45%)	NR	Median 3
		HTO	41	58.4 ± 8.4	20 (48.8%)	NR	Median 3

Abbreviations: BMI, body mass index; HTO, high tibial osteotomy; LOE, level of evidence; NR, not reported; SD, standard deviation.

### Study demographics

All 17 studies were comparative studies comparing HTO plus arthroscopy to HTO alone. The studies differed in the concomitant arthroscopic procedures performed. Six studies [2, 9, 21, 23, 26, 30] evaluated HTO with microfracture, comparing it to HTO alone. Three studies [13, 14, 27] compared HTO with microdrilling to HTO alone. Two studies [12, 15] compared HTO with meniscal centralization of meniscal extrusions to HTO alone. One study [23] compared HTO with abrasion arthroplasty and HTO, with this study also having a HTO plus microfracture group as a third arm. Another study [27] compared HTO with abrasion arthroplasty to HTO alone, with a third arm investigating HTO with microdrilling. Two studies [5, 16] assessed HTO with meniscectomy, both partial and subtotal, compared to HTO. Four studies [4, 28, 34, 35] assessed HTO with mixed arthroscopic interventions compared to HTO. No included study compared HTO plus meniscal repair with HTO alone.

### Risk of bias and study quality

The two RCTs were assessed using RoB 2 (Table S2). One study [26] was judged to raise some concerns, and the other study [35] was rated as high risk of bias. Fifteen non‐randomized comparative studies were assessed using MINORS. The mean MINORS score was 14.9 ± 2.3, ranging from 10 to 18 (Table S3).

### Patient demographics

Across the 17 studies, the pooled sample included 1271 knees, comprising 663 treated with HTO plus arthroscopy and 608 treated with HTO. Where reported, the weighted mean age was 57.0 years (range: 41.4–65.0 years) in the HTO plus arthroscopy cohorts and 55.1 years (39.3–64.5 years) in the HTO cohorts. The weighted proportion of male patients was 35.8% and 33.1%, respectively. Weighted mean body mass index (BMI) was 26.8 kg/m^2^ (25.1–31.0 kg/m^2^) and 27.0 kg/m^2^ (25.5–28.6 kg/m^2^), respectively. Weighted mean FU was 32.9 months (12–69.3 months) and 31.0 months (12–67.4 months), respectively.

### HTO + microfracture versus HTO

#### PROMs

Lysholm scores were reported in two studies (*n* = 169) [26, 30] (Table 2). With HTO plus microfracture, the weighted mean Lysholm score improved from 60.5 preoperatively to 84.7 postoperatively (mean improvement +24.2). With HTO alone, the weighted mean Lysholm score improved from 58.2 preoperatively to 89.3 postoperatively (+31.1). One study [30] reported a significant postoperative difference favouring HTO alone over HTO plus microfracture (92.3 vs. 86.3, *p* = 0.002). In contrast, the other study [26] found no significant postoperative difference between the groups (80.3 vs. 78.3, *p* = 0.425).

**Table 2:** Summary of PROMs and functional outcomes in included studies.

Reference	Minimum FU	Concomitant arthroscopic procedure(s)	Outcome	HTO + arthroscopy pre (mean ± SD)	HTO + arthroscopy post (mean ± SD)	HTO pre (mean ± SD)	HTO post (mean ± SD)	Post *p* value
Matsunaga et al. [23]	5 years	Abrasion arthroplasty	JOA Knee score	52.4 ± 6	87 ± 7.8	49.8 ± 6.8	88.9 ± 4.8	NR
Cho et al. [4]	NR	Mixed arthroscopy	KSS Knee	55.3	86.4	58.2	81.5	NR
Shen et al. [28]	2 years	Mixed arthroscopy	VAS	6	1.8	6	4	0.028
			Lysholm	53	93	54	80	0.031
			HSS	60	84	62	78	0.045
Kim et al. [16]	2 years	Meniscectomy	ROM, °	128.3 ± 7.7	133.2 ± 6	128 ± 8.7	128.5 ± 7.7	0.011
			VAS	6.7 ± 1	1.5 ± 0.9	6.8 ± 1	1.7 ± 0.7	0.845
			KSS Knee	56.9 ± 5.5	88.8 ± 5.6	57.9 ± 5.1	89.3 ± 5.4	0.943
			KSS Function	53.6 ± 5.3	89.4 ± 4.9	53.5 ± 5.2	89.8 ± 5	0.646
Horita et al. [12]	2 years	Meniscal centralization	KOOS Symptoms	61.3 ± 15.8	80.5 ± 16.2	62.6 ± 15.5	81.1 ± 13.3	0.999
			KOOS Pain	69.3 ± 19.1	81 ± 17.8	62.6 ± 22.4	80 ± 17.1	0.755
			KOOS ADL	68.6 ± 15.6	86.9 ± 12.7	69.3 ± 18.1	84.9 ± 15.5	0.735
			KOOS Sport/Recreation	33.6 ± 18.1	72.8 ± 21.1	33.1 ± 25.1	56.1 ± 27.5	0.039
			KOOS QoL	32.1 ± 16	69.4 ± 19.1	32.7 ± 20.7	62.6 ± 22.4	0.313
Katagiri et al. [15]	2 years	Meniscal centralization	Flexion, °	140	145	140	145	NR
			IKDC	NR	71.8	NR	65.2	0.22
			KOOS Pain	NR	82.9	NR	79.9	0.58
			KOOS Symptoms	NR	81.8	NR	78.8	0.58
			KOOS ADL	NR	90.3	NR	90.8	0.89
			KOOS Sport/Recreation	NR	67.1	NR	63.3	0.67
			KOOS QoL	NR	67.9	NR	68.1	0.97
			Lysholm	NR	94.1	NR	90.9	0.22
Jung et al. [13]	2 years	Microdrilling	VAS	62	17	58	25	NR
			Lysholm	47.5	77	44	71.7	NR
			IKDC	32.8	58.1	32.8	55	NR
			KOOS Pain	47	77.7	41.8	71.8	NR
			KOOS Symptoms	50	72.2	43.6	67.1	NR
			KOOS ADL	56	81.7	54	75.9	NR
			KOOS Sport/Recreation	20.8	47.3	19.6	45	NR
			KOOS QoL	26	56	27.8	53.7	NR
			Kujala	43	70.4	37.8	60.4	NR
Aygün et al. [2]	5 years	Microfracture	KOOS	61.23 ± 7.46	88.27 ± 2.75	63.71 ± 6.87	81.42 ± 2.89	<0.001
			KOOS Pain	NR	82.69 ± 2.71	NR	73.88 ± 2.23	<0.001
			KOOS Symptoms	NR	81.65 ± 2.74	NR	81.71 ± 2.76	0.938
			KOOS ADL	NR	87.08 ± 3.35	NR	80.29 ± 2.35	<0.001
			KOOS Sport/Recreation	NR	79.54 ± 3.59	NR	79.08 ± 3.34	0.895
			KOOS QoL	NR	80.62 ± 3.82	NR	80.67 ± 3.07	0.557
			VAS	8 ± 0.75	2.27 ± 0.6	7.71 ± 0.69	3.54 ± 0.66	<0.001
			ROM, °	102.5 ± 5.87	118.46 ± 6.13	103.54 ± 5.21	102.71 ± 5.1	<0.001
Ferruzzi et al. [9]	11 years	Microfracture	HSS	35	59	37	71	NR
			WOMAC	35	62	34	73	NR
Lee et al. [21]	2 years	Microfracture	WOMAC	39.8 ± 13.2	9.2 ± 6.1	39.5 ± 7.8	10.2 ± 7.8	0.513
			KSS Knee	53.7 ± 17	89.1 ± 10.7	52.7 ± 14.3	88.3 ± 10.8	0.745
			KSS Function	59.5 ± 15.5	88.3 ± 10.8	59.8 ± 9	86.1 ± 12.3	0.634
			Further Flexion, °	132.1 ± 7.2	134.9 ± 7.2	134 ± 4.8	136 ± 6.6	0.492
			Flexion contracture, °	2.5 ± 1.4	2.2 ± 1.2	1.8 ± 2.8	1.8 ± 1.6	0.403
Matsunaga et al. [23]	5 years	Microfracture	JOA knee score	52.5 ± 8.7	85.6 ± 7.4	49.8 ± 6.8	88.9 ± 4.8	NR
Souza et al. [30]	2 years	Microfracture	Lysholm	65.5 ± 5.2	86.3 ± 12.8	60.8 ± 7	92.3 ± 8.8	0.002
Pascale et al. [26]	5 years	Microfracture	Lysholm	46.5 ± 12.3	80.3 ± 8	48.8 ± 10.4	78.3 ± 7.7	0.425
Zhang et al. [34]	1 year	Mixed arthroscopy	KSS (median, IQR)	50.0 (25.0)	60.0 (40.0)	57.5 (41.0)	80.0 (10.0)	NR
			HSS (median, IQR)	62.0 (10.0)	74.0 (12.0)	55.0 (18.0)	80.0 (13.0)	NR
			WOMAC (median, IQR)	43.5 (12.0)	20.0 (16.0)	44.0 (12.0)	11.0 (12.0)	NR
Zhao et al. [35]	6 months	Mixed arthroscopy	HSS	NR	87.84 ± 6.23	NR	83.28 ± 4.13	<0.001
			KSS Knee	NR	89.76 ± 4.32	NR	79.92 ± 4.82	<0.001
			VAS	NR	0.89 ± 0.19	NR	1.28 ± 0.15	<0.001
Choe et al. [5]	4 years	Partial meniscectomy	HSS	72.9 ± 12	89.3 ± 9.1	78.7 ± 7.3	90.4 ± 8.1	0.843
			KSS Knee	76 ± 11	90.9 ± 11.2	79.3 ± 7.3	88.6 ± 9.8	0.814
			KSS Functional	65.6 ± 7.3	86.9 ± 13.5	70.6 ± 11.5	87.1 ± 12.5	0.188
Jung et al. [14]	2 years	Microdrilling	KSS Function	66.5 ± 14.3	92.8 ± 10	66.8 ± 9.1	92.2 ± 8	0.806
			KSS Knee	67.3 ± 8.2	91.2 ± 6.4	63.7 ± 13.9	92.5 ± 5.3	0.389
Choe et al. [5]	4 years	Subtotal meniscectomy	HSS	72.1 ± 8.5	88.6 ± 9.1	NR	NR	NR
			KSS Knee	79.9 ± 8.4	91.3 ± 10.3	NR	NR	NR
			KSS Functional	68.9 ± 14.5	80.7 ± 13	NR	NR	NR

*Note*: The table shows mean ± SD unless otherwise stated. Preoperative and postoperative values are presented where available. Postoperative *p* values represent between‐group comparisons at final FU, where reported.

Abbreviations: ADL, activities of daily living; FU, follow‐up; HSS, Hospital for Special Surgery score; HTO, high tibial osteotomy; IKDC, International Knee Documentation Committee score; IQR, interquartile range; JOA, Japanese Orthopaedic Association; KOOS, Knee injury and Osteoarthritis Outcome Score; KSS, Knee Society Score; NR, not reported; n.s., non‐significant; QoL, quality of life; PROMs, patient‐reported outcome measures; ROM, range of motion; SD, standard deviation; VAS, visual analogue scale; WOMAC, Western Ontario and McMaster Universities Osteoarthritis Index.

Western Ontario McMaster Universities Osteoarthritis Index (WOMAC) scores were reported by two studies (*n* = 125) [9, 21] (Table 2). Both studies reported improvements in WOMAC scores but had differing defined directions of improvement (higher = better in one study [9] and lower = better in the other study [21]). In one study [9], the mean WOMAC score improved from 35 preoperatively to 62 postoperatively with microfracture, compared with 34–73 in the HTO alone cohort, with mean improvements of 27 and 39, respectively. In the other study [21], the mean WOMAC score improved from 39.8 to 9.2 with HTO plus microfracture, compared with 39.5–10.2 with HTO alone. Mean improvements were 30.6 and 29.3. One study [21] reported no significant postoperative difference between HTO plus microfracture and HTO, with respect to WOMAC scores (*p* = 0.513).

#### Radiographic outcomes

Hip–knee–ankle (HKA) angle was reported by two studies (*n* = 125) [9, 21] (Table 3). In one study [9], the median HKA angle improved from 9° varus preoperatively to 4° valgus postoperatively in the HTO plus microfracture cohort, compared with 8° varus to 4° valgus with HTO. Median changes were 13° and 12° valgus, respectively. In the other study [21], the mean HKA angle improved from 7.0° varus to 2.3° valgus postoperatively with microfracture, compared with 6.2° varus to 1.9° valgus in the HTO cohort. Mean changes were 9.3° and 8.1° valgus, respectively. This study found no significant difference in postoperative HKA angle between the groups (*p* = 0.270).

**Table 3:** Summary of radiographic outcomes in included studies.

Reference	Minimum FU	Concomitant arthroscopic procedure(s)	Radiographic outcome	HTO + arthroscopy pre (mean ± SD)	HTO + arthroscopy post (mean ± SD)	HTO pre (mean ± SD)	HTO post (mean ± SD)	Post *p* value
Matsunaga et al. [23]	5 years	Abrasion arthroplasty	FTA, °	185 (varus 5) ± 4.3	167 (valgus 13) ± 2.9	185 (varus 5) ± 5.4	166 (valgus 14) ± 3.3	NR
Cho et al. [4]	NR	Mixed arthroscopy	TFA, °	Varus 8.9°	Valgus 7.8°	Varus 9.0°	Valgus 6.4°	NR
Shen et al. [28]	2 years	Mixed arthroscopy	HKA, °	169	185	171	176	0.016
			MPTA, °	77	90	78	82	0.025
Kim et al. [16]	2 years	Meniscectomy	mFTA, °	8.8 ± 3.6	−3.2 ± 1.9	7.6 ± 3.3	−3 ± 3.3	0.55
			PTS, °	5.4 ± 3.2	7.4 ± 3.4	5.6 ± 3.1	7.9 ± 4.1	0.681
Horita et al. [12]	2 years	Meniscal centralization	WBLR (%)	29.8 ± 7.7	51.9 ± 8.7	28.2 ± 14.1	61.4 ± 7.6	<0.001
			HKA, °	4.2 ± 2	−0.7 ± 2.3	4.4 ± 3.6	−3.1 ± 1.6	<0.001
			mLDFA, °	87.7 ± 1.5	87.6 ± 2.2	87.5 ± 2.1	87.5 ± 1.7	0.261
			MPTA, °	85.2 ± 1.8	90.7 ± 2	85.3 ± 2.2	92.6 ± 1.9	0.003
			JLCA, °	1.7 ± 1.4	2 ± 1.4	1.9 ± 2.9	2.6 ± 2.2	0.488
Katagiri et al. [15]	2 years	Meniscal centralization	JLCA, °	4.2	3.3	3.9	3.5	NR
			FTA, °	181.9	172.6	181.9	174.2	0.24
			HKA, °	8.5	−1.6	7.7	−0.3	0.13
			WBLR (%)	13.4	55.3	10.9	45.9	0.02
Jung et al. [13]	2 years	Microdrilling	WBLR (%)	16.7 ± 11.5	68 ± 8.7	20.6 ± 15.1	67.4 ± 10.5	0.947
			MPTA, °	85.7 ± 2.4	94.9 ± 2.1	85.4 ± 2.2	94.8 ± 2.2	0.697
Ferruzzi et al. [9]	11 years	Microfracture	HKA (median), °	9	4	8	4	NR
Lee et al. [21]	2 years	Microfracture	WBLR (%)	19.1 ± 11.7	58.6 ± 7.2	22 ± 12.3	57.5 ± 6.2	0.06
			HKA, °	7 ± 3.1	−2.3 ± 1.4	6.2 ± 2.7	−1.9 ± 1.6	0.27
			JLCA, °	3.1 ± 1.7	1.9 ± 1.7	2.8 ± 1.4	2.1 ± 2.1	0.705
Matsunaga et al. [23]	5 years	Microfracture	FTA, °	185 (varus 4) ± 4.3	168 (valgus 12) ± 3.6	185 (varus 5) ± 5.4	166 (valgus 14) ± 3.3	
Pascale et al. [26]	5 years	Microfracture	FTA, °	178.5 ± 1.5	184.5 ± 0.9	178.2 ± 1.6	184.6 ± 0.9	NS
Zhang et al. [34]	1 year	Mixed arthroscopy (median, IQR)	MPTA, °	84, IQR 4	93, IQR 8	84, IQR 3	93, IQR 4	NR
			JLCA, °	3, IQR 1	1, IQR 1	3, IQR 3	3, IQR 2	NR
			FTA, °	6, IQR 6	2, IQR 5	9, IQR 4	1, IQR 3	NR
			HKA, °	174, IQR 7	179, IQR 4	171, IQR 5	180, IQR 1	NR
			WBLR, %	24.9, IQR 22.14	53.6, IQR 30.36	14.5, IQR 16.32	53.4, IQR 17.76	NR
Zhao et al. [35]	6 months	Mixed arthroscopy	HKA, °	NR	5.13 (valgus) ± 0.22	NR	5.09 (valgus) ± 0.16	0.349
			MPTA, °	NR	97.12 ± 2.07	NR	96.89 ± 1.81	0.593
			PTS, °	NR	8.03 ± 0.76	NR	7.92 ± 0.36	0.404
Choe et al. [5]	4 years	Partial meniscectomy	HKA, °	8 ± 2.6	−2.1 ± 2.7	6.6 ± 2.1	2 ± 2.7	0.947
Jung et al. [14]	2 years	Subchondral drilling	mTFA, °	−4.5 ± 2.4	3.5 ± 1.4	−5 ± 2.2	3.2 ± 1.9	0.471
Choe et al. [5]	4 years	Subtotal meniscectomy	HKA, °	6.3 ± 3.2	−1.9 ± 2.5	NR	NR	NR

*Note*: The table shows mean ± SD unless otherwise stated. Preoperative and postoperative values are presented where available. Postoperative *p* values represent between‐group comparisons at final FU, where reported. Alignment conventions are presented as reported by the original studies.

Abbreviations: FTA, femorotibial angle; FU, follow‐up; HKA, hip–knee–ankle angle; HTO, high tibial osteotomy; IQR, interquartile range; JLCA, joint line convergence angle; mFTA, mechanical femorotibial angle; mLDFA, mechanical lateral distal femoral angle; MPTA, medial proximal tibial angle; mTFA, mechanical tibiofemoral angle; NR, not reported; PTS, posterior tibial slope; SD, standard deviation; TFA, tibiofemoral angle; WBLR, weight‐bearing line ratio.

Femorotibial angle (FTA) was reported by two studies (*n* = 103) [23, 26] (Table 3). In one study [23], mean FTA changed from 185° preoperatively to 168° postoperatively in the HTO plus microfracture cohort (mean correction: 17° valgus), compared with 185°–166° in the HTO alone cohort (mean correction: 19° valgus). In the other study [26], mean FTA changed from 178.5° preoperatively to 184.5° postoperatively in the microfracture cohort (mean change: 6° valgus), compared with 178.2°–184.6° in the HTO alone cohort (mean change: 6.4° valgus).

#### Second‐look arthroscopy or cartilage status

Second‐look arthroscopy outcomes were reported by two studies (*n* = 150) [21, 23]. One study [23] assessed cartilage repair 1 year postoperatively with the Outerbridge classification. There was no significant difference in femoral or tibial condylar cartilage repair between HTO plus microfracture and HTO alone. Another study [21] assessed pre‐ to postoperative cartilage change using the International Cartilage Repair Society (ICRS) grading system. Improvement in medial femoral condyle ICRS grade was observed in 75.4% of patients undergoing HTO plus microfracture compared with 53.3% undergoing HTO alone (*p* = 0.014). The study also reported higher postoperative Magnetic Resonance Observation of Cartilage Repair Tissue (MOCART) scores after HTO plus microfracture compared with HTO (41.8 ± 18.6 vs. 31.8 ± 19.8, *p* = 0.023), driven primarily by improved osteotomy defect filling (*p* < 0.001).

#### Complications

Complications were reported by one study [9], reporting stiffness in 2 of 18 patients (11.1%) treated with HTO plus microfracture, compared with 0 of 20 patients (0%) treated with HTO. No delayed unions or superficial infections occurred in the microfracture cohort, whereas the HTO cohort had one delayed union (1/20, 5.0%) and no superficial infections. Conversion to total knee arthroplasty (TKA) occurred in 3 of 18 patients (16.7%) after HTO plus microfracture and 1 of 20 patients (5.0%) after HTO.

### HTO + microdrilling versus HTO

#### PROMs

PROMs after HTO with microdrilling were reported by two studies (*n* = 121) [13, 14] (Table 2). In one study [13], visual analogue scale (VAS) pain scores improved from 62 preoperatively to 17 postoperatively after HTO plus microdrilling and from 58 to 25 after HTO alone, with mean improvements of 45 and 33. Lysholm scores improved from 47.5 to 77.0 after microdrilling and from 44.0 to 71.7 after HTO (mean improvements: 29.5 and 27.7). Knee injury and Osteoarthritis Outcome Score (KOOS) and Knee Society Score (KSS) improvements were broadly similar between groups (Table 2).

#### Radiographic outcomes

Radiographic outcomes were reported by the same two studies (Table 3). In one study [13], weight‐bearing line ratio (WBLR) moved from 16.7% to 68.0% after HTO plus microdrilling and 20.6% to 67.4% after HTO, with no significant postoperative between‐group difference (*p* = 0.947). Medial proximal tibial angle (MPTA) increased from 85.7° to 94.9° after HTO plus microdrilling and from 85.4° to 94.8° after HTO, with no significant postoperative between‐group difference (*p* = 0.697).

#### Second‐look arthroscopy or cartilage status

Second‐look arthroscopy and structural cartilage outcomes were reported by three studies (*n* = 183). One study [14] found no significant difference in grade II fibrocartilage formation (100% vs. 94%, *p* = 0.492) or fibrocartilage maturation (10% vs. 3%, *p* = 0.354). Another study [27], using arthroscopic Collins staging and histological assessment, reported that microdrilling groups demonstrated significantly better cartilage regeneration than HTO groups (*p* < 0.001), with regenerated tissue described as thicker and covering a wider area. In contrast, one study [13] reported significantly better trochlear cartilage status, using ICRS grading, after HTO plus microdrilling compared with HTO (93.3% vs. 36.7%, *p* < 0.001).

#### Complications

Complications were reported by one study [14], which reported correction failure in 2 of 30 patients (6.7%) treated with HTO plus microdrilling and 5 of 31 patients (16.1%) with HTO.

### HTO + abrasion arthroplasty versus HTO

#### PROMs

One study [23] reported Japanese Orthopaedic Association (JOA) Knee Scores after HTO with abrasion arthroplasty compared to HTO (*n* = 88), with similar mean improvements from baseline (+34.6 vs. +39.1) (Table 2).

#### Radiographic outcomes

Radiographic outcomes were reported by the same study (*n* = 88) (Table 3). In the abrasion arthroplasty cohort, mean FTA changed from 185° preoperatively (5° varus) to 167° postoperatively (13° valgus), with a mean change of 18° valgus. In the HTO cohort, mean FTA changed from 185° to 166° (mean change: 19° valgus).

#### Second‐look arthroscopy or cartilage status

Second‐look arthroscopy findings were reported by two studies (*n* = 151) [23, 27]. One study [27] found that the abrasion arthroplasty group demonstrated significantly better cartilage regeneration than HTO alone (*p* < 0.001). One study [23] reported improved tibial and superior femoral condyle repair after HTO plus abrasion arthroplasty compared with HTO (*p* < 0.005). No complications were reported in these two studies.

### HTO + meniscal centralization versus HTO

#### PROMs

PROMs after HTO with meniscal centralization were reported by two studies (*n* = 87) [12, 15] (Table 2). For KOOS Pain, the weighted postoperative mean was 81.9 after HTO plus meniscal centralization and 80.0 after HTO. Neither study found a significant postoperative between‐group difference (Table 2). For KOOS Sport, the weighted postoperative mean was 70.1 after meniscal centralization and 59.2 after HTO alone. One study [12] reported a significantly higher postoperative KOOS Sport after HTO plus meniscal centralization (72.8 vs. 56.1, *p* = 0.039), whereas another [15] found no significant difference (67.1 vs. 63.3, *p* = 0.67).

#### Radiographic outcomes

Radiographic outcomes were reported by the same two studies [12, 15] (Table 3). HKA improved in both groups, with a weighted mean change from 6.2° varus preoperatively to 1.1° valgus postoperatively after meniscal centralization, and 5.8° varus to 1.9° valgus after HTO. One study [12] reported a significant postoperative difference in HKA between groups (0.7° valgus vs. 3.1° valgus, *p* < 0.001), whereas the other study [15] found no significant difference (*p* = 0.13). WBLR also improved in both groups, with a weighted mean change from 22.1% to 53.5% after meniscal centralization and from 20.8% to 54.8% after HTO.

#### Second‐look arthroscopy

Second‐look arthroscopy findings were reported by one study (*n* = 48) [12], reporting greater tibial and femoral cartilage structural improvement with meniscal centralization compared with HTO alone.

#### Complications

Complications were reported by one study [15], which reported one superficial surgical site infection among 21 patients treated with HTO plus meniscal centralization (4.8%), and none among 18 patients treated with HTO (0%).

### HTO + meniscectomy versus HTO

PROMs following HTO with meniscectomy were reported by two studies (*n* = 138) [5, 16] (Table 2). The weighted mean KSS Knee score improved from 67.1 to 89.9 after HTO plus meniscectomy and from 63.7 to 89.1 with HTO. Both studies found no significant difference in postoperative KSS Knee. The weighted mean KSS Function score improved from 60.3 to 86.4 after HTO plus meniscectomy and from 58.2 to 89.1 with HTO alone, with no significant difference in postoperative KSS Function in both studies.

#### Radiographic outcomes

Radiographic outcomes were reported by one study (*n* = 79) [16] (Table 3), which found no significant postoperative between‐group difference in mFTA (*p* = 0.550).

#### Complications

Complications were reported by two studies [5, 16]. One study [16] reported one deep infection among 55 patients treated with HTO plus meniscectomy (1.8%), compared with no deep infections among 24 patients treated with HTO. One study [5] reported that Type 1 lateral hinge fractures occurred in 4 of 20 patients (20.0%) who underwent partial meniscectomy, with no Type 2 or Type 3 fractures. In the subtotal meniscectomy cohort, Type 1 fractures occurred in 5 of 30 patients (16.7%), and Type 2 fractures occurred in 1 of 30 patients (3.3%), with no Type 3 fractures. With HTO alone, Type 1 lateral hinge fractures occurred in two of nine patients (22.2%), with no Type 2 or Type 3 fractures reported.

### HTO + mixed arthroscopic interventions versus HTO

#### PROMs

In the HTO plus mixed arthroscopy subgroup, the arthroscopic procedures performed varied across studies and included debridement, meniscectomy, meniscal repair and/or microfracture. KSS Knee scores were reported by two studies (*n* = 127) (Table 2). One study [4] reported improvement from 55.3 preoperatively to 86.4 postoperatively after HTO plus mixed arthroscopy, compared with 58.2–81.5 after HTO. One study [35] reported significantly higher postoperative KSS Knee scores after HTO plus mixed arthroscopy (89.76 vs. 79.92, *p* < 0.001).

VAS pain scores were reported by two studies (*n* = 190) (Table 2). One study [28] reported improvement from 6.0 to 1.8 after HTO plus mixed arthroscopy and 6.0 to 4.0 after HTO, favouring mixed arthroscopy at final FU (*p* = 0.028). Another study [35] similarly reported significantly lower postoperative VAS scores after HTO plus mixed arthroscopy compared with HTO (0.89 vs. 1.28, *p* < 0.001).

#### Radiographic outcomes

Three studies reported HKA angle (*n* = 353) (Table 3). One study [35] reported no significant postoperative difference in HKA angle between mixed arthroscopy and HTO (5.13° vs. 5.09° valgus, *p* = 0.349). One study [34] reported median HKA angle improvement from 174° to 179° after mixed arthroscopy and 171°–180° after HTO. One study [28] reported a greater postoperative HKA angle after mixed arthroscopy compared with HTO (185° vs. 176°, *p* = 0.016).

MPTA was reported by three studies (*n* = 353). Two studies [34, 35] found no significant postoperative difference in MPTA between HTO plus mixed arthroscopy and HTO alone. Conversely, one study [28] reported significantly greater postoperative MPTA after HTO plus mixed arthroscopy (90° vs. 82°, *p* = 0.025).

#### Complications

Complications were reported by one study (*n* = 23) [4], which reported one haematoma in the mixed arthroscopy cohort (7.7%), with no cases of peroneal palsy, delayed union or infection. In the HTO cohort, two peroneal nerve palsies (20%), one delayed union (10%) and one infection (10%) were reported, with no haematomas.

## DISCUSSION

The principal finding of this review is that both HTO with concomitant arthroscopy and HTO alone were generally associated with improvements in PROMs, functional outcomes and radiographic alignment. However, no specific chondral or meniscal treatment demonstrated consistent additional clinical benefit in HTO, with respect to PROMs or radiographic outcomes. Microfracture, microdrilling, abrasion arthroplasty and meniscal centralization were associated with improved cartilage findings upon second‐look arthroscopy, but these structural improvements did not consistently translate into superior PROMs, functional outcomes or radiographic alignment. These findings suggest that correction of varus malalignment via HTO is likely the major driver of symptomatic improvement in patients, while the incremental clinical benefit of routinely treating intra‐articular pathology remains uncertain.

Microfracture and microdrilling involve penetrating the subchondral bone with a perforating awl, Kirschner wire or drill to induce marrow bleeding and fibrin clot formation within the chondral defect, allowing marrow‐derived mesenchymal cells to contribute to fibrocartilaginous repair [8]. Similarly, abrasion arthroplasty involves debriding worn cartilage into subchondral bone using an abrader, thereby stimulating fibrocartilage formation [8]. Although adding these marrow‐stimulating procedures was associated with improved cartilage findings in some studies [13, 21, 23, 27], PROMs, such as Lysholm, WOMAC, KSS and KOOS, did not consistently favour the combined approach [13, 21, 26, 30]. This may reflect that HTO itself creates a favourable environment for cartilage healing by unloading pressure on the medial compartment; as such, concomitant arthroscopy could potentially be producing a redundant effect. In support of this, previous systematic reviews reported that both HTO with or without cartilage procedures similarly promote cartilage regeneration [11]. However, prior reviews are limited because they pooled heterogeneous procedures. Nonetheless, based on current evidence, the structural cartilage improvement conferred by arthroscopic interventions during HTO may not provide clinically meaningful improvement in patient symptoms, pending higher‐quality evidence.

Furthermore, meniscectomy performed during HTO did not consistently improve KSS outcomes compared with HTO alone [5, 16]. However, this finding is vulnerable to confounding by indication, because patients who underwent meniscectomy had meniscal pathology, whereas the patients who underwent HTO alone did not. Therefore, the absence of superior PROMs does not imply that symptomatic or mechanically obstructive meniscal tears should never be treated during HTO. Rather, current evidence does not support routine meniscectomy as a universally beneficial adjunct. This distinction is important, particularly in the context of the broader knee arthroscopy literature, where arthroscopic debridement and partial meniscectomy have shown limited benefit for degenerative meniscal tears compared with nonoperative treatment [17, 29].

In the present review, adding meniscal centralization to HTO was associated with improvements in cartilage status and selected KOOS domains, but other PROM and radiographic findings were inconsistent [12, 15]. Meniscal centralization involves suturing an extruded meniscus to the rim of the tibial plateau to restore meniscal position, improving its ability to distribute load and reduce peak tibiofemoral contact stresses [24]. Wider literature suggests meniscal centralization can reduce meniscal extrusion and may improve clinical or biomechanical outcomes [3], but such evidence is not specific to combined HTO and meniscal centralization. Therefore, meniscal centralization remains a promising but incompletely defined adjunct.

Four studies [4, 28, 34, 35] compared HTO alone with HTO combined with a mixture of concomitant arthroscopic procedures, including chondral and meniscal procedures performed according to intraoperative findings or patient‐specific pathology. This mixed arthroscopy subgroup appeared to present the most supportive findings for concomitant arthroscopy during HTO; some studies reported superior VAS, KSS, Hospital for Special Surgery (HSS), HKA angle and MPTA outcomes compared to HTO alone [28, 35]. While this subgroup is the least internally interpretable, it may best reflect current real‐world practice, where surgeons use variable interventions to treat incidental intra‐articular pathology based on the individual patient. Nevertheless, these mixed groups do not isolate the effect of any specific arthroscopic procedure, and the findings cannot be interpreted as evidence supporting routine arthroscopy for all patients undergoing HTO. Instead, the evidence may suggest that selected patients can benefit from targeted pathology‐directed arthroscopic treatment, but clearer indication‐based algorithms are lacking and required.

Complication reporting was limited and heterogeneous across the included studies, which precludes conclusions regarding comparative safety. Overall, few major complications were reported, but most studies were underpowered to detect uncommon adverse events and rarely reported operative time, reoperation, revision HTO, conversion to arthroplasty and cost. This is important because even if arthroscopy adds little direct morbidity, it may increase operative burden, procedural complexity and resource use [10].

Several limitations of this review should be acknowledged. The included studies were both clinically and methodologically heterogeneous, with variation in patient selection, arthroscopic procedures, indications, clinical outcome measures and FU duration, all of which precluded quantitative analysis. Although statistically significant between‐group differences in PROMs were reported, most included studies did not report the proportion of patients achieving minimal clinically important differences (MCID), precluding assessment of the clinical meaningfulness of reported PROM improvements. Additionally, most studies were retrospective and non‐randomized, creating unavoidable confounding by indication. This was particularly relevant for meniscectomy, meniscal centralization and mixed arthroscopy studies, where baseline pathology likely differed between the treatment groups. In addition, several studies lacked granular reporting or characterization of intra‐articular pathology, meniscal tear morphology, cartilage lesion size or location and rehabilitation protocols. This limited the ability to determine whether specific pathologies or patient subgroups may benefit most from concomitant arthroscopy during HTO.

The findings of this review identify clear priorities for future research. Procedure‐specific RCTs with long‐term FU are needed, with eligibility restricted to patients sharing similar baseline pathology. For example, studies should compare HTO plus meniscectomy versus HTO alone in patients with defined meniscal tear patterns and HTO plus microfracture or microdrilling versus HTO in patients with comparable chondral lesions. A pragmatic RCT investigating HTO plus pathology‐directed mixed arthroscopy versus HTO alone is also needed, with transparent reporting of procedure‐specific outcomes. Importantly, future studies should report the proportion of patients reaching MCID or Patient Acceptable Symptom States (PASS) to facilitate assessment of the clinical meaningfulness of any observed improvements.

## CONCLUSION

Current comparative evidence does not demonstrate a consistent clinical benefit to adding concomitant arthroscopy to treat intra‐articular chondral or meniscal pathology during valgus‐producing HTO. Microfracture, microdrilling, abrasion arthroplasty and meniscal centralization may improve cartilage appearance, but these structural improvements do not consistently translate into superior PROMs, functional outcomes or radiographic correction. These findings suggest that realignment via HTO may be the major driver of symptomatic improvement, and the incremental benefit of treating intra‐articular pathology remains unclear. Current evidence does not support routine arthroscopy for all patients undergoing HTO; however, selected pathology‐directed arthroscopic treatment may still be appropriate pending more conclusive evidence. Future procedure‐specific comparative RCTs are needed to determine whether the investigated chondral or meniscal arthroscopic interventions provide clinically meaningful benefit beyond HTO alone.

## AUTHOR CONTRIBUTIONS

All authors contributed to study design, data collection, analysis and manuscript preparation.

## CONFLICT OF INTEREST STATEMENT

The authors declare no conflict of interest.

## ETHICS STATEMENT

The authors have nothing to report.

## Supporting information

Supplementary Table 1. Search Strategies.

Supplementary Table 2. RoB 2 Risk of Bias Assessment.

Supplementary Table 3. MINORS Quality Assessment.

## Data Availability

The data that support the findings of this study are available in the supporting information of this article.
