# Supplementary material for: Concomitant arthroscopy during valgus‐producing high tibial osteotomy does not demonstrate consistent additional clinical benefit despite improved cartilage findings: A systematic review of comparative studies
Source: J Exp Orthop. 2026 Sep 7;13(3):e70903. doi: 10.1002/jeo2.70903 (PMC13549371; doi:10.1002/jeo2.70903)
Supplement: Supplementary file 1 [file JEO2-13-e70903-s001.docx]

**Supplementary Table 1.** Search strategies

**MEDLINE via Ovid <1946 to 17 May 2026>**

1. exp Osteotomy/ (43,419)
2. exp Tibia/ (42,437)
3. (“high tibial osteotomy” or HTO or “medial opening wedge” or “opening wedge osteotomy” or “open wedge osteotomy” or “closed wedge osteotomy” or “closing wedge osteotomy” or “proximal tibial osteotomy” or “tibial osteotomy”).mp. (5,609)
4. 1 and 2 (4,941)
5. 3 or 4 (8,033)
6. exp Arthroscopy/ (31,707)
7. (“arthroscop*” or “meniscectomy” or “partial meniscectomy” or “meniscal debridement” or “meniscus debridement” or “meniscal trimming” or “meniscus trimming” or “meniscal repair” or “meniscus repair” or “meniscal suture” or “meniscus suture” or “root repair” or “meniscal root repair” or “meniscus root repair” or “medial meniscus posterior root” or “MMPRT” or “chondroplasty” or “chondral debridement” or “cartilage debridement” or microfracture or “abrasion arthroplasty” or “drilling” or “bone marrow stimulation” or “cartilage repair” or “cartilage restoration” or “cartilage regeneration” or “second-look arthroscopy” or lavage or synovectomy or “loose body” or “loose bodies”).mp. (156,788)
8. 6 or 7 (156,867)
9. (“osteoarthritis” or “knee osteoarthritis” or varus or “varus knee” or “medial compartment” or “medial compartment osteoarthritis” or “medial compartment overload” or gonarthrosis).mp. (147,544)
10. 5 and 8 and 9 (578)

**Embase via Ovid <1974 to 17 May 2026>**

1. (“high tibial osteotomy” or “HTO” or “medial opening wedge” or “opening wedge osteotomy” or “open wedge osteotomy” or “closed wedge osteotomy” or “closing wedge osteotomy” or “proximal tibial osteotomy” or “tibial osteotomy”).mp. (260927)
2. (“arthroscop*” or “meniscectomy” or “partial meniscectomy” or “meniscal debridement” or “meniscus debridement” or “meniscal trimming” or “meniscus trimming” or “meniscal repair” or “meniscus repair” or “meniscal suture” or “meniscus suture” or “root repair” or “meniscal root repair” or “meniscus root repair” or “medial meniscus posterior root” or “MMPRT” or “chondroplasty” or “chondral debridement” or “cartilage debridement” or “microfracture” or “abrasion arthroplasty” or drilling or “bone marrow stimulation” or “cartilage repair” or “cartilage restoration” or “cartilage regeneration” or “second-look arthroscopy” or “lavage” or “synovectomy” or “loose body” or “loose bodies”).mp. (239475)
3. 1 and 2 (1121)

**Cochrane Library <inception to 17 May 2026>**

1. (“high tibial osteotomy” OR “HTO” OR “medial opening wedge” OR “opening wedge osteotomy” OR “open wedge osteotomy” OR “closed wedge osteotomy” OR “closing wedge osteotomy” OR “proximal tibial osteotomy” OR “tibial osteotomy”):ti,ab,kw (433)
2. (“arthroscop*” or “meniscectomy” or “partial meniscectomy” or “meniscal debridement” or “meniscus debridement” or “meniscal trimming” or “meniscus trimming” or “meniscal repair” or “meniscus repair” or “meniscal suture” or “meniscus suture” or “root repair” or “meniscal root repair” or “meniscus root repair” or “medial meniscus posterior root” or “MMPRT” or “chondroplasty” or “chondral debridement” or “cartilage debridement” or microfracture or “abrasion arthroplasty” or drilling or “bone marrow stimulation” or “cartilage repair” or “cartilage restoration” or “cartilage regeneration” or “second-look arthroscopy” or “lavage” or synovectomy or “loose body” or “loose bodies”):ti,ab,kw (11337)
3. (“osteoarthritis” or “knee osteoarthritis” or “varus” or “varus knee” or “medial compartment” or “medial compartment osteoarthritis” or “medial compartment overload” or “gonarthrosis”):ti,ab,kw (29026)
4. #1 AND #2 AND #3 (72)
