# Supplementary material for: Concomitant arthroscopy during valgus‐producing high tibial osteotomy does not demonstrate consistent additional clinical benefit despite improved cartilage findings: A systematic review of comparative studies
Source: J Exp Orthop. 2026 Sep 7;13(3):e70903. doi: 10.1002/jeo2.70903 (PMC13549371; doi:10.1002/jeo2.70903)
Supplement: Supplementary file 2 [file JEO2-13-e70903-s002.docx]

**Supplementary Table 3.** MINORS Quality Assessment.

|  | **Criteria** | | | | | | | | | | | | | |  |
| --- | --- | --- | --- | --- | --- | --- | --- | --- | --- | --- | --- | --- | --- | --- | --- |
| **References** | **1** | **2** | **3** | **4** | **5** | **6** | **7** | **8** |  | **9** | **10** | **11** | **12** | **TOTAL MINORS SCORE** | **Maximum** **possible score** |
| Aygun et al., 2024 | 2 | 1 | 2 | 2 | 0 | 2 | 1 | 0 |  | 2 | 2 | 2 | 2 | 18 | 24 |
| Choe et al., 2023 | 2 | 1 | 0 | 2 | 1 | 2 | 0 | 0 |  | 1 | 2 | 1 | 1 | 13 | 24 |
| Cho et al., 1991 | 2 | 1 | 0 | 2 | 0 | 1 | 1 | 0 |  | 2 | 0 | 1 | 0 | 10 | 24 |
| Ferruzzi et al., 2014 | 2 | 1 | 0 | 2 | 0 | 2 | 2 | 0 |  | 2 | 2 | 1 | 2 | 16 | 24 |
| Horita et al., 2025 | 2 | 2 | 0 | 2 | 1 | 2 | 1 | 2 |  | 2 | 0 | 2 | 2 | 18 | 24 |
| Jung et al., 2025 | 2 | 2 | 0 | 2 | 1 | 2 | 0 | 0 |  | 2 | 0 | 2 | 2 | 15 | 24 |
| Jung et al., 2015 | 2 | 1 | 0 | 2 | 1 | 2 | 2 | 0 |  | 2 | 0 | 2 | 2 | 16 | 24 |
| Katagiri et al., 2023 | 2 | 1 | 0 | 2 | 1 | 2 | 0 | 0 |  | 1 | 1 | 1 | 2 | 13 | 24 |
| Kim et al., 2017 | 2 | 1 | 0 | 2 | 0 | 2 | 0 | 0 |  | 1 | 0 | 2 | 2 | 12 | 24 |
| Lee et al., 2019 | 2 | 1 | 0 | 2 | 1 | 2 | 0 | 0 |  | 2 | 2 | 2 | 2 | 16 | 24 |
| Matsunaga et al., 2007 | 2 | 1 | 1 | 2 | 0 | 2 | 2 | 0 |  | 2 | 0 | 1 | 2 | 15 | 24 |
| Schultz and Gobel, 1999 | 2 | 1 | 2 | 2 | 0 | 1 | 0 | 0 |  | 2 | 2 | 1 | 2 | 15 | 24 |
| Shen et al., 2024 | 2 | 0 | 0 | 2 | 0 | 2 | 1 | 0 |  | 2 | 2 | 1 | 1 | 13 | 24 |
| Souza et al., 2018 | 2 | 1 | 0 | 2 | 0 | 2 | 2 | 0 |  | 2 | 2 | 1 | 2 | 16 | 24 |
| Zhang et al., 2024 | 2 | 1 | 0 | 2 | 2 | 2 | 1 | 0 |  | 2 | 2 | 2 | 2 | 18 | 24 |

*Note.* MINORS = Methodological Index for Non-randomized Studies

MINORS are scored from 0 (*not reported*), 1 (*reported but inadequate*), and 2 (*reported and adequate*).

The global maximum score for non-comparative studies is 16.

The global maximum score for comparative studies is 24.

**MINORS Criteria**

**1.** **A clearly stated aim:** *the question addressed should be both specific and relevant.*

**2. Inclusion of consecutive patients:** *all potentially fit patients have been included.*

**3. Prospective collection of data:** *data collected according to prior-established protocol before the beginning of the study.*

**4.** **Endpoints appropriate to the aim of study:** *unambiguous endpoint criteria, on an intention-to-treat basis.*

**5**. **Unbiased assessment of study endpoint:** *blind evaluation of objective endpoints, double blinding of subjective endpoints, with stated reasoning in the case of non-blinding.*

**6. Follow-up period appropriate for the aim of study:** *follow-up period of at least 12 months to assess main endpoint(s) and* *possible complications.*

**7. Loss to follow-up less than 5%:** *all patients included in follow-up with reporting of follow-up loss of less than 5% throughout study’s duration.*

**8. Prospective calculation of study size:** *size of detectable differences, calculation of 95% confidence interval, power estimate, and level of statistical significance calculations performed.*

**9. An adequate control-group:** *having an adequate and relevant control group or gold-standard diagnostic test or therapeutic intervention, in accordance with published data.*

**10. Contemporary groups:** *control group (if-any) and studied group should be managed within the same* *time period.*

**11. Baseline equivalence of groups:** *groups should be similar regarding criteria other than studied endpoints, ideally absence of confounding factors that could bias interpretation.*

**12. Adequate statistical analyses:** *statistical analyses in accordance with study-type, including calculation of confidence interval or relative risk.*
