# Supplementary material for: Concomitant arthroscopy during valgus‐producing high tibial osteotomy does not demonstrate consistent additional clinical benefit despite improved cartilage findings: A systematic review of comparative studies
Source: J Exp Orthop. 2026 Sep 7;13(3):e70903. doi: 10.1002/jeo2.70903 (PMC13549371; doi:10.1002/jeo2.70903)
Supplement: Supplementary file 3 [file JEO2-13-e70903-s003.docx]

**Supplementary Table 2.** RoB 2 Risk of Bias Assessment.

| **Study** | **D1: Randomization process** | **D2: Deviations from intended interventions** | **D3: Missing outcome data** | **D4: Measurement of the outcome** | **D5: Selection of the reported result** | **Overall risk of bias** |
| --- | --- | --- | --- | --- | --- | --- |
| Pascale et al., 2011 | Some concerns | Low | Low | Low | Some concerns | Some concerns |
| Zhao et al., 2022 | Some concerns | Some concerns | Low | High | Some concerns | High |
